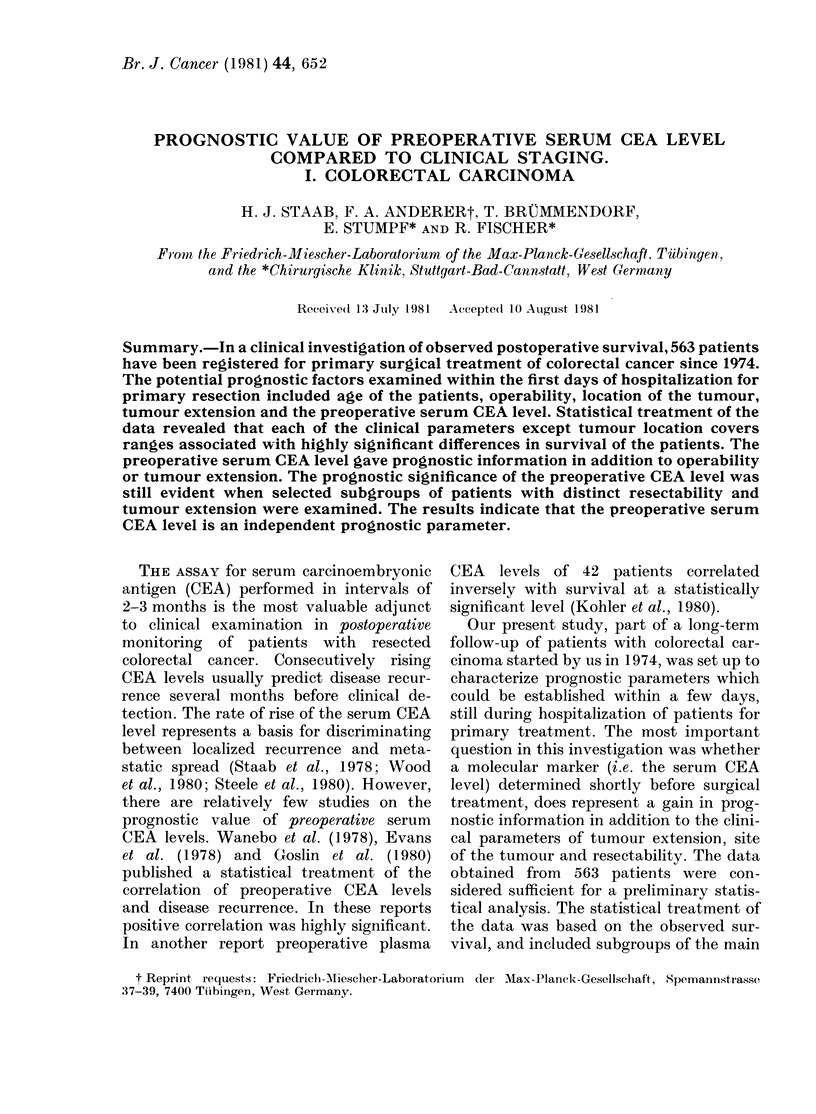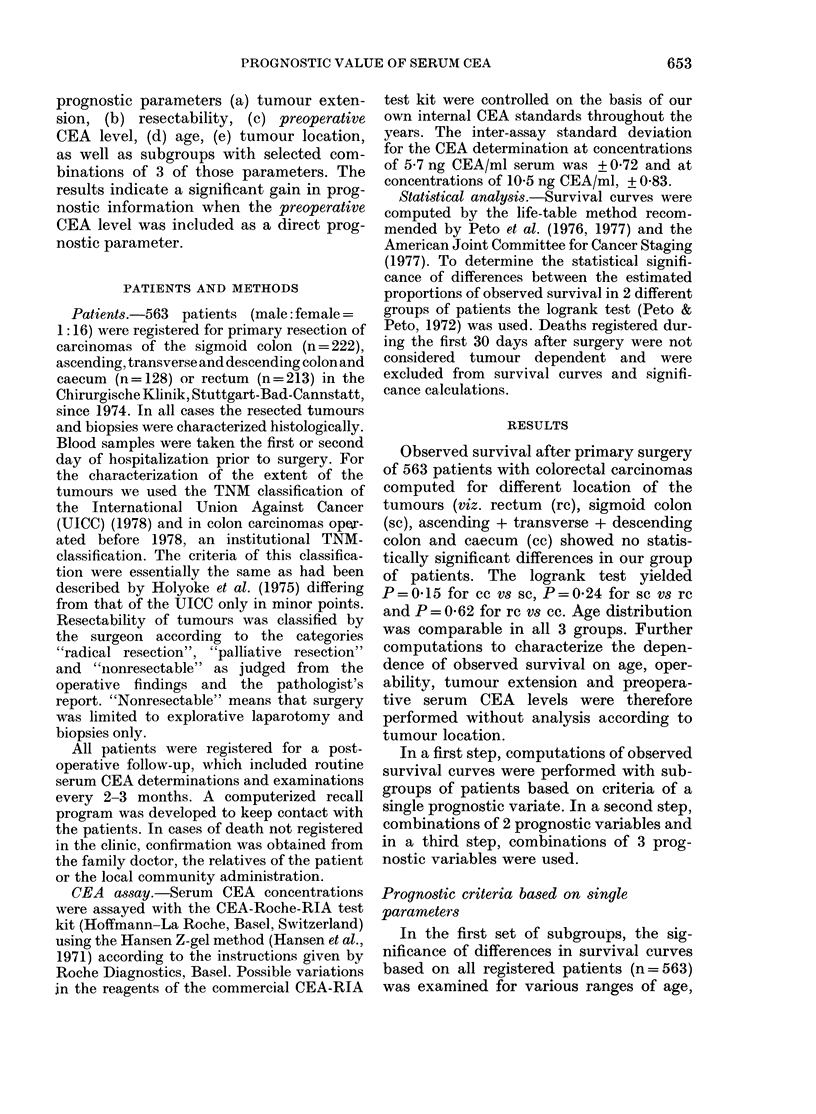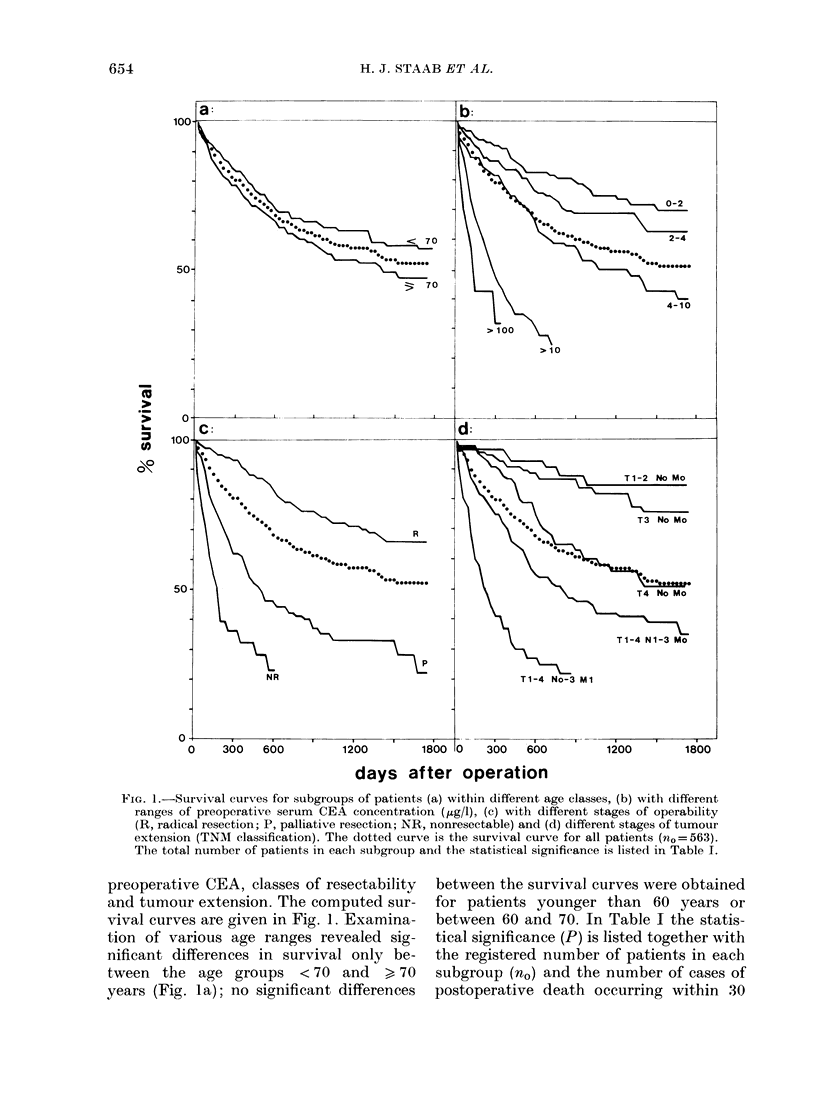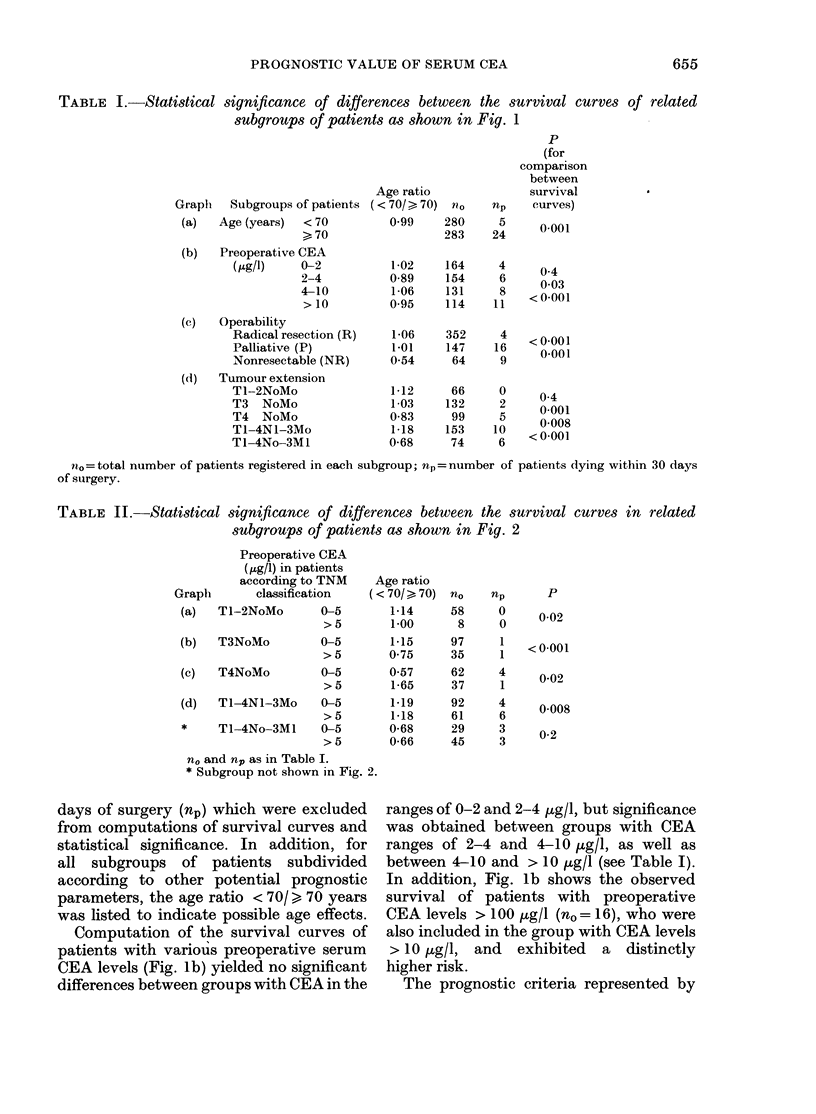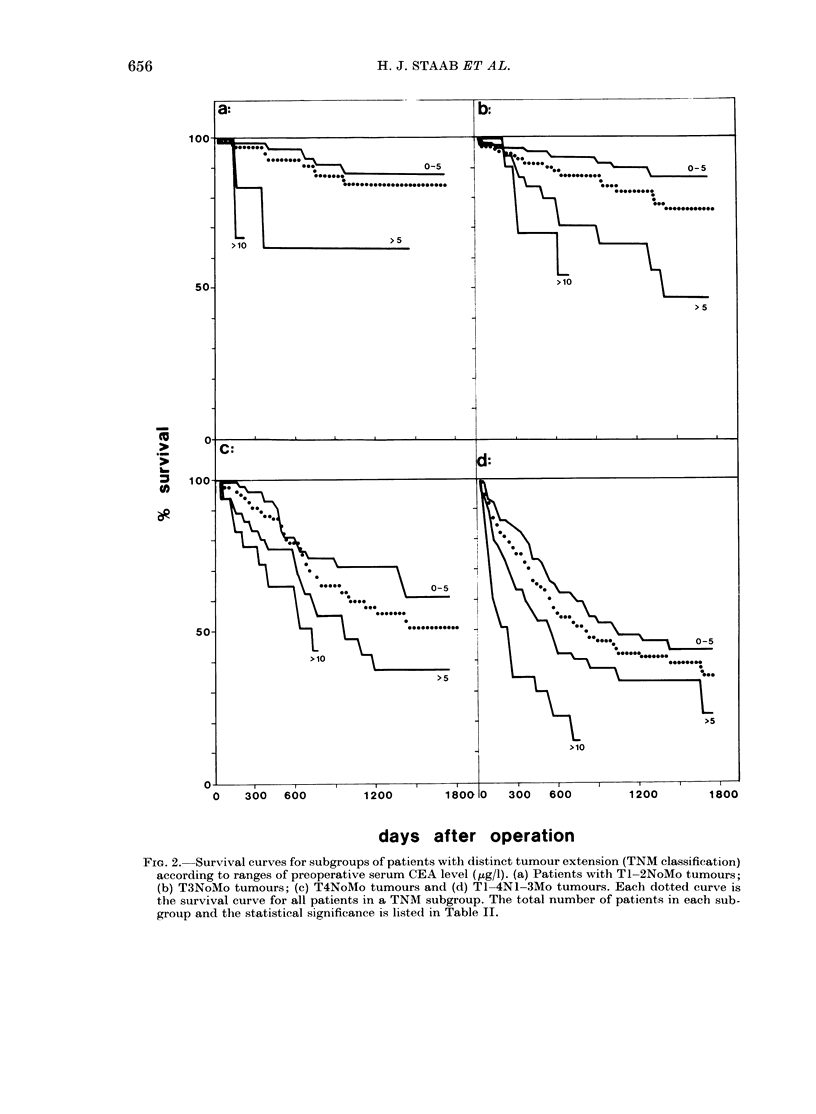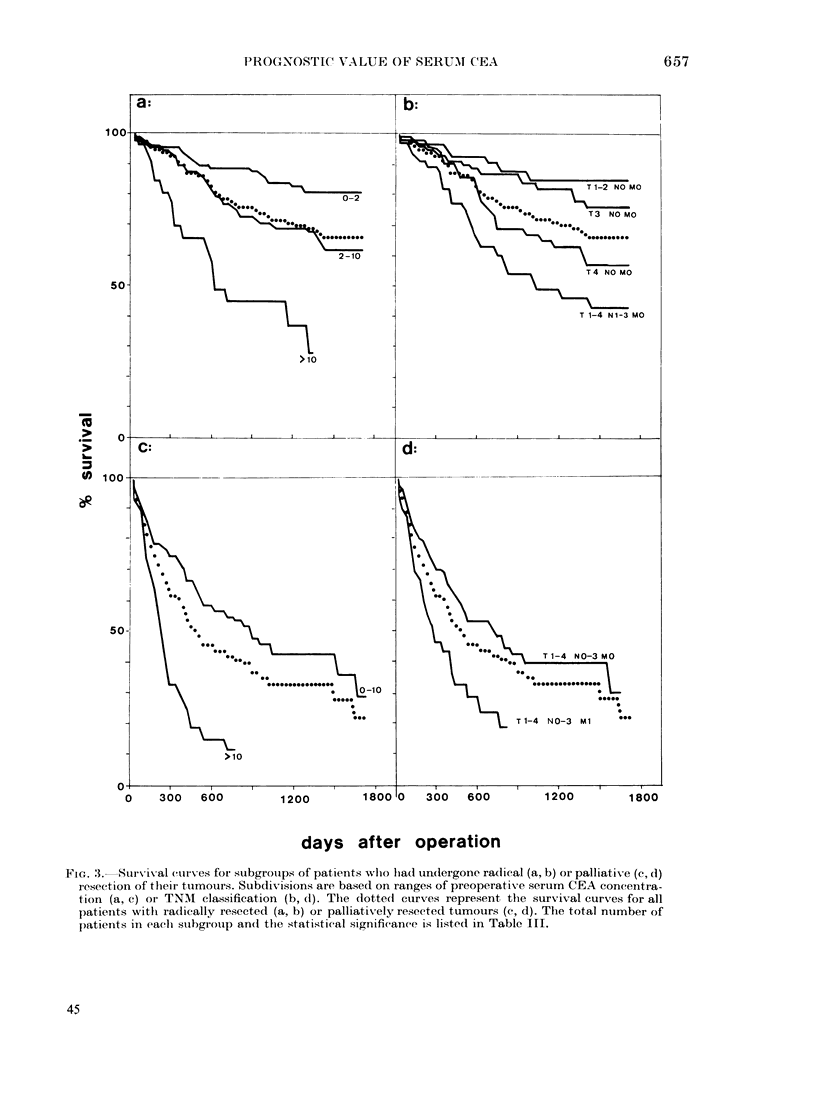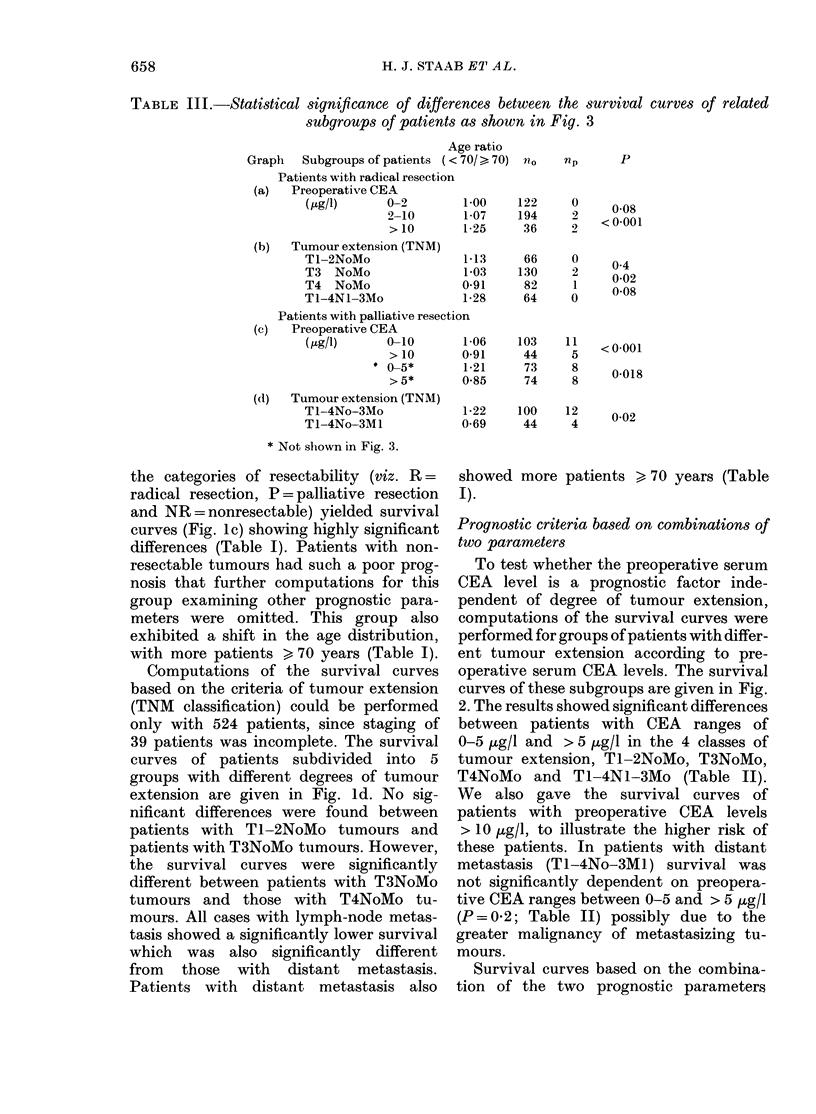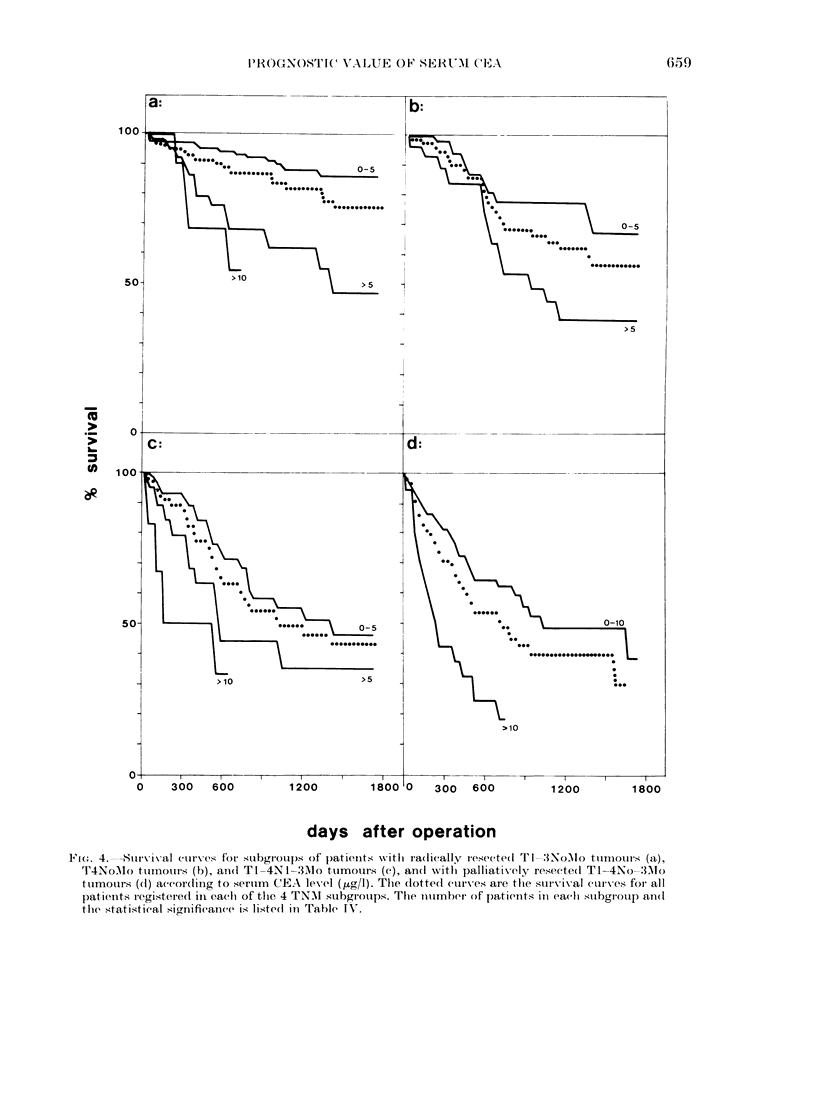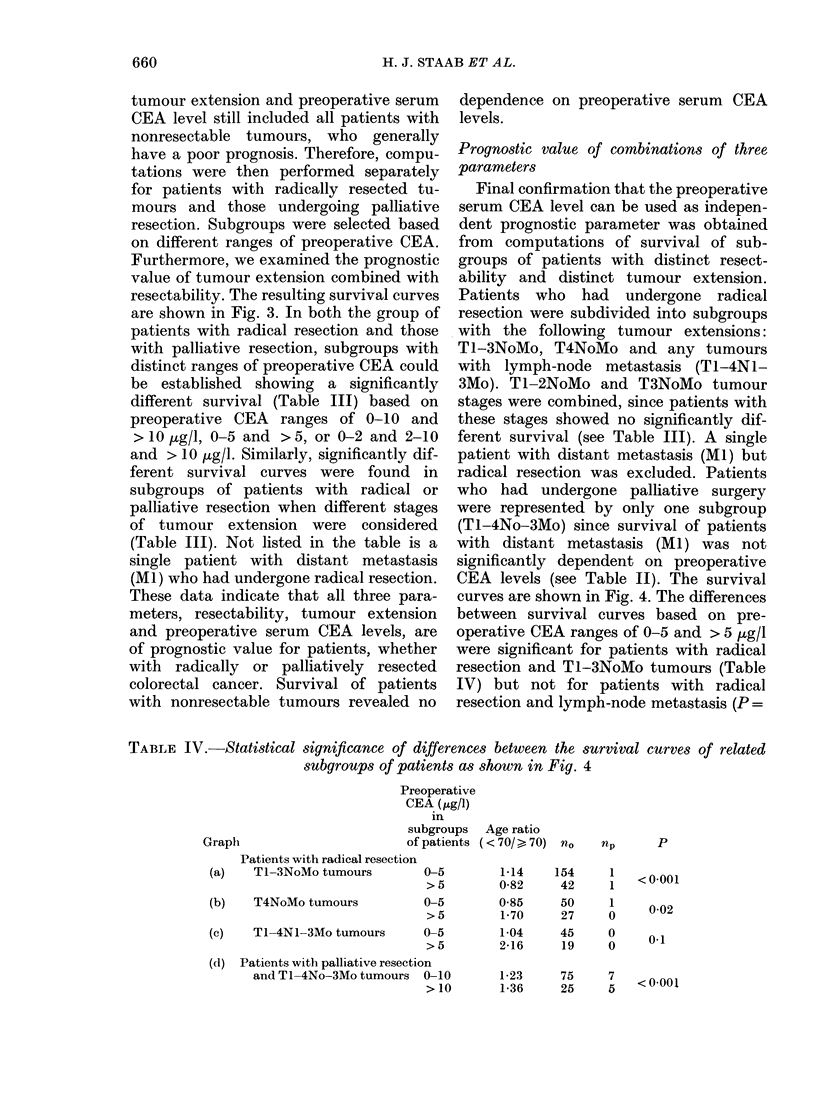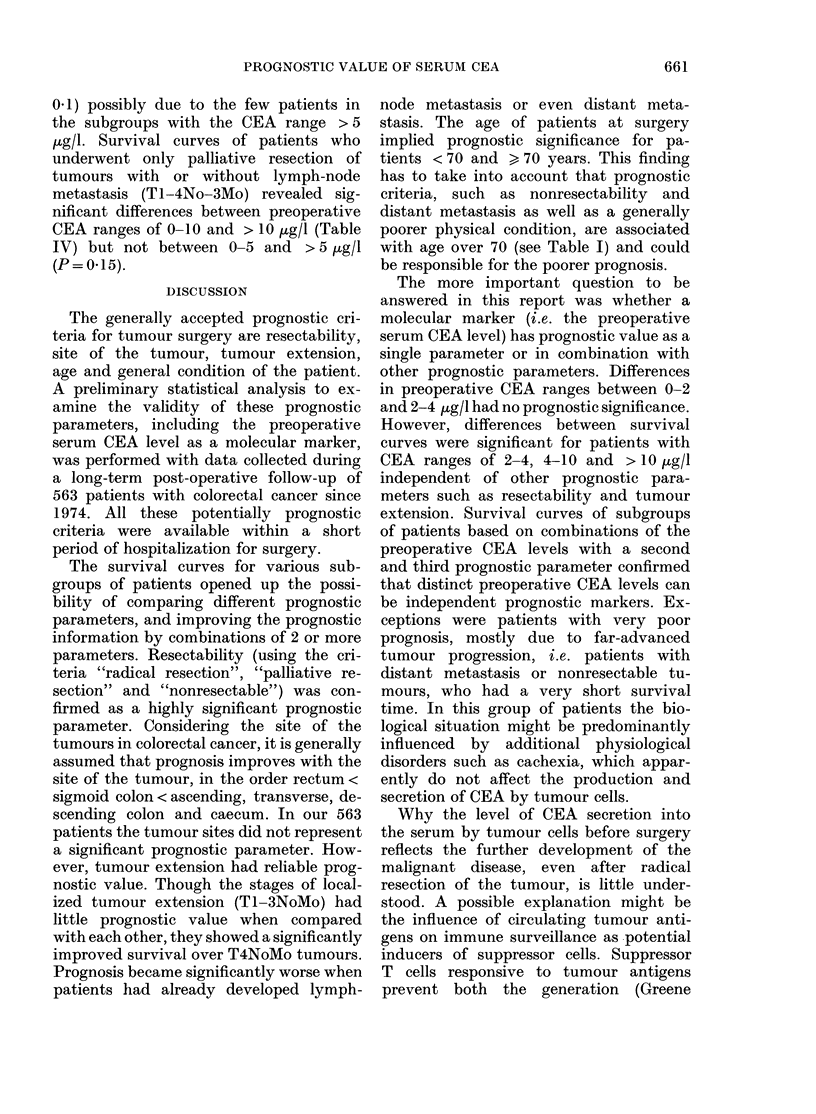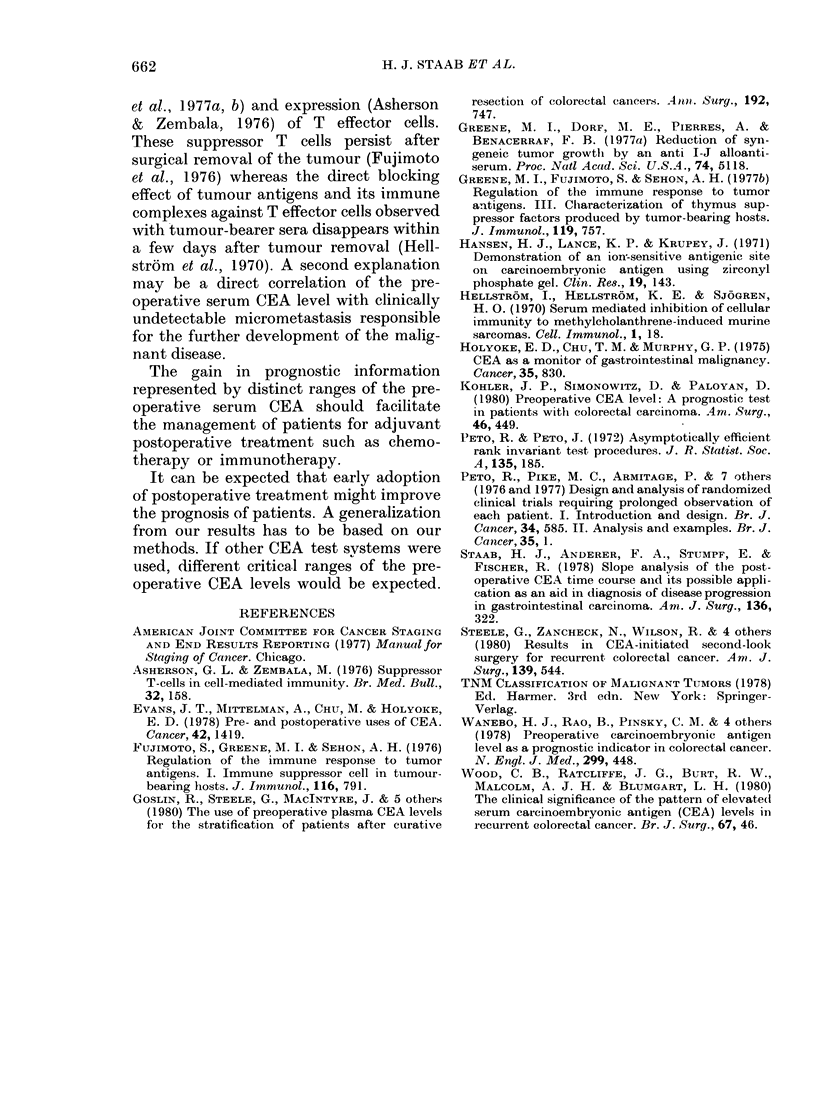# Prognostic value of preoperative serum CEA level compared to clinical staging. I. Colorectal carcinoma.

**DOI:** 10.1038/bjc.1981.250

**Published:** 1981-11

**Authors:** H. J. Staab, F. A. Anderer, T. Brümmendorf, E. Stumpf, R. Fischer

## Abstract

In a clinical investigation of observed postoperative survival, 563 patients have been registered for primary surgical treatment of colorectal cancer since 1974. The potential prognostic factors examined within the first days of hospitalization for primary resection included age of the patients, operability, location of the tumour, tumour extension and the preoperative serum CEA level. Statistical treatment of the data revealed that each of the clinical parameters except tumour location covers ranges associated with highly significant differences in survival of the patients. The preoperative serum CEA level gave prognostic information in addition to operability or tumour extension. The prognostic significance of the preoperative CEA level was still evident when selected subgroups of patients with distinct resectability and tumour extension were examined. The results indicate that the preoperative serum CEA level is an independent prognostic parameter.


*Br. J. Cancer* (1981) **44**, 652

### PROGNOSTIC VALUE OF PREOPERATIVE SERUM CEA LEVEL COMPARED TO CLINICAL STAGING. I. COLORECTAL CARCINOMA

H. J. STAAB, F. A. ANDERER†, T. BRÜMMENDORF, E. STUMPF* AND R. FISCHER*

*From the Friedrich-Miescher-Laboratorium of the Max-Planck-Gesellschaft, Tübingen, and the *Chirurgische Klinik, Stuttgart-Bad-Cannstatt, West Germany*

Received 13 July 1981 Accepted 10 August 1981

**Summary.—In a clinical investigation of observed postoperative survival, 563 patients have been registered for primary surgical treatment of colorectal cancer since 1974. The potential prognostic factors examined within the first days of hospitalization for primary resection included age of the patients, operability, location of the tumour, tumour extension and the preoperative serum CEA level. Statistical treatment of the data revealed that each of the clinical parameters except tumour location covers ranges associated with highly significant differences in survival of the patients. The preoperative serum CEA level gave prognostic information in addition to operability or tumour extension. The prognostic significance of the preoperative CEA level was still evident when selected subgroups of patients with distinct resectability and tumour extension were examined. The results indicate that the preoperative serum CEA level is an independent prognostic parameter.**

THE ASSAY for serum carcinoembryonic antigen (CEA) performed in intervals of 2–3 months is the most valuable adjunct to clinical examination in *postoperative* monitoring of patients with resected colorectal cancer. Consecutively rising CEA levels usually predict disease recurrence several months before clinical detection. The rate of rise of the serum CEA level represents a basis for discriminating between localized recurrence and metastatic spread (Staab *et al.*, 1978; Wood *et al.*, 1980; Steele *et al.*, 1980). However, there are relatively few studies on the prognostic value of *preoperative* serum CEA levels. Wanebo *et al.* (1978), Evans *et al.* (1978) and Goslin *et al.* (1980) published a statistical treatment of the correlation of preoperative CEA levels and disease recurrence. In these reports positive correlation was highly significant. In another report preoperative plasma CEA levels of 42 patients correlated inversely with survival at a statistically significant level (Kohler *et al.*, 1980).

Our present study, part of a long-term follow-up of patients with colorectal carcinoma started by us in 1974, was set up to characterize prognostic parameters which could be established within a few days, still during hospitalization of patients for primary treatment. The most important question in this investigation was whether a molecular marker (*i.e.* the serum CEA level) determined shortly before surgical treatment, does represent a gain in prognostic information in addition to the clinical parameters of tumour extension, site of the tumour and resectability. The data obtained from 563 patients were considered sufficient for a preliminary statistical analysis. The statistical treatment of the data was based on the observed survival, and included subgroups of the main

† Reprint requests: Friedrich-Miescher-Laboratorium der Max-Planck-Gesellschaft, Spemannstrasse 37–39, 7400 Tübingen, West Germany.

prognostic parameters (a) tumour extension, (b) resectability, (c) *preoperative* CEA level, (d) age, (e) tumour location, as well as subgroups with selected combinations of 3 of those parameters. The results indicate a significant gain in prognostic information when the *preoperative* CEA level was included as a direct prognostic parameter.

### PATIENTS AND METHODS

*Patients.*—563 patients (male:female = 1:16) were registered for primary resection of carcinomas of the sigmoid colon (n = 222), ascending, transverse and descending colon and caecum (n = 128) or rectum (n = 213) in the Chirurgische Klinik, Stuttgart-Bad-Cannstatt, since 1974. In all cases the resected tumours and biopsies were characterized histologically. Blood samples were taken the first or second day of hospitalization prior to surgery. For the characterization of the extent of the tumours we used the TNM classification of the International Union Against Cancer (UICC) (1978) and in colon carcinomas operated before 1978, an institutional TNM-classification. The criteria of this classification were essentially the same as had been described by Holyoke *et al.* (1975) differing from that of the UICC only in minor points. Resectability of tumours was classified by the surgeon according to the categories "radical resection", "palliative resection" and "nonresectable" as judged from the operative findings and the pathologist's report. "Nonresectable" means that surgery was limited to explorative laparotomy and biopsies only.

All patients were registered for a post-operative follow-up, which included routine serum CEA determinations and examinations every 2–3 months. A computerized recall program was developed to keep contact with the patients. In cases of death not registered in the clinic, confirmation was obtained from the family doctor, the relatives of the patient or the local community administration.

*CEA assay.*—Serum CEA concentrations were assayed with the CEA-Roche-RIA test kit (Hoffmann–La Roche, Basel, Switzerland) using the Hansen Z-gel method (Hansen *et al.*, 1971) according to the instructions given by Roche Diagnostics, Basel. Possible variations in the reagents of the commercial CEA-RIA test kit were controlled on the basis of our own internal CEA standards throughout the years. The inter-assay standard deviation for the CEA determination at concentrations of 5·7 ng CEA/ml serum was ±0·72 and at concentrations of 10·5 ng CEA/ml, ±0·83.

*Statistical analysis.*—Survival curves were computed by the life-table method recommended by Peto *et al.* (1976, 1977) and the American Joint Committee for Cancer Staging (1977). To determine the statistical significance of differences between the estimated proportions of observed survival in 2 different groups of patients the logrank test (Peto & Peto, 1972) was used. Deaths registered during the first 30 days after surgery were not considered tumour dependent and were excluded from survival curves and significance calculations.

### RESULTS

Observed survival after primary surgery of 563 patients with colorectal carcinomas computed for different location of the tumours (*viz.* rectum (rc), sigmoid colon (sc), ascending + transverse + descending colon and caecum (cc) showed no statistically significant differences in our group of patients. The logrank test yielded $P = 0{\cdot}15$ for cc *vs* sc, $P = 0{\cdot}24$ for sc *vs* rc and $P = 0{\cdot}62$ for rc *vs* cc. Age distribution was comparable in all 3 groups. Further computations to characterize the dependence of observed survival on age, operability, tumour extension and preoperative serum CEA levels were therefore performed without analysis according to tumour location.

In a first step, computations of observed survival curves were performed with subgroups of patients based on criteria of a single prognostic variate. In a second step, combinations of 2 prognostic variables and in a third step, combinations of 3 prognostic variables were used.

### *Prognostic criteria based on single parameters*

In the first set of subgroups, the significance of differences in survival curves based on all registered patients (n = 563) was examined for various ranges of age,

FIG. 1.—Survival curves for subgroups of patients (a) within different age classes, (b) with different ranges of preoperative serum CEA concentration (μg/l), (c) with different stages of operability (R, radical resection; P, palliative resection; NR, nonresectable) and (d) different stages of tumour extension (TNM classification). The dotted curve is the survival curve for all patients ($n_0 = 563$). The total number of patients in each subgroup and the statistical significance is listed in Table I.

preoperative CEA, classes of resectability and tumour extension. The computed survival curves are given in Fig. 1. Examination of various age ranges revealed significant differences in survival only between the age groups $< 70$ and $\geq 70$ years (Fig. 1a); no significant differences between the survival curves were obtained for patients younger than 60 years or between 60 and 70. In Table I the statistical significance ($P$) is listed together with the registered number of patients in each subgroup ($n_0$) and the number of cases of postoperative death occurring within 30

TABLE I.—*Statistical significance of differences between the survival curves of related subgroups of patients as shown in Fig.* 1

| Graph | Subgroups of patients | | Age ratio ($<70/\geqslant 70$) | $n_o$ | $n_p$ | *P* (for comparison between survival curves) |
|---|---|---|---|---|---|---|
| (a) | Age (years) | $<70$ | 0·99 | 280 | 5 | 0·001 |
| | | $\geqslant 70$ | | 283 | 24 | |
| (b) | Preoperative CEA | | | | | |
| | ($\mu$g/l) | 0–2 | 1·02 | 164 | 4 | 0·4 |
| | | 2–4 | 0·89 | 154 | 6 | 0·03 |
| | | 4–10 | 1·06 | 131 | 8 | $<$0·001 |
| | | $>10$ | 0·95 | 114 | 11 | |
| (c) | Operability | | | | | |
| | Radical resection (R) | | 1·06 | 352 | 4 | $<$0·001 |
| | Palliative (P) | | 1·01 | 147 | 16 | 0·001 |
| | Nonresectable (NR) | | 0·54 | 64 | 9 | |
| (d) | Tumour extension | | | | | |
| | T1–2NoMo | | 1·12 | 66 | 0 | 0·4 |
| | T3 NoMo | | 1·03 | 132 | 2 | 0·001 |
| | T4 NoMo | | 0·83 | 99 | 5 | 0·008 |
| | T1–4N1–3Mo | | 1·18 | 153 | 10 | $<$0·001 |
| | T1–4No–3M1 | | 0·68 | 74 | 6 | |

$n_o$ = total number of patients registered in each subgroup; $n_p$ = number of patients dying within 30 days of surgery.

TABLE II.—*Statistical significance of differences between the survival curves in related subgroups of patients as shown in Fig.* 2

| Graph | Preoperative CEA ($\mu$g/l) in patients according to TNM classification | | Age ratio ($<70/\geqslant 70$) | $n_o$ | $n_p$ | *P* |
|---|---|---|---|---|---|---|
| (a) | T1–2NoMo | 0–5 | 1·14 | 58 | 0 | 0·02 |
| | | $>5$ | 1·00 | 8 | 0 | |
| (b) | T3NoMo | 0–5 | 1·15 | 97 | 1 | $<$0·001 |
| | | $>5$ | 0·75 | 35 | 1 | |
| (c) | T4NoMo | 0–5 | 0·57 | 62 | 4 | 0·02 |
| | | $>5$ | 1·65 | 37 | 1 | |
| (d) | T1–4N1–3Mo | 0–5 | 1·19 | 92 | 4 | 0·008 |
| | | $>5$ | 1·18 | 61 | 6 | |
| * | T1–4No–3M1 | 0–5 | 0·68 | 29 | 3 | 0·2 |
| | | $>5$ | 0·66 | 45 | 3 | |

$n_o$ and $n_p$ as in Table I.
* Subgroup not shown in Fig. 2.

days of surgery ($n_p$) which were excluded from computations of survival curves and statistical significance. In addition, for all subgroups of patients subdivided according to other potential prognostic parameters, the age ratio $<70/\geqslant 70$ years was listed to indicate possible age effects.

Computation of the survival curves of patients with various preoperative serum CEA levels (Fig. 1b) yielded no significant differences between groups with CEA in the ranges of 0–2 and 2–4 $\mu$g/l, but significance was obtained between groups with CEA ranges of 2–4 and 4–10 $\mu$g/l, as well as between 4–10 and $>10$ $\mu$g/l (see Table I). In addition, Fig. 1b shows the observed survival of patients with preoperative CEA levels $>100$ $\mu$g/l ($n_o = 16$), who were also included in the group with CEA levels $>10$ $\mu$g/l, and exhibited a distinctly higher risk.

The prognostic criteria represented by

FIG. 2.—Survival curves for subgroups of patients with distinct tumour extension (TNM classification) according to ranges of preoperative serum CEA level (μg/l). (a) Patients with T1–2NoMo tumours; (b) T3NoMo tumours; (c) T4NoMo tumours and (d) T1–4N1–3Mo tumours. Each dotted curve is the survival curve for all patients in a TNM subgroup. The total number of patients in each subgroup and the statistical significance is listed in Table II.

FIG. 3.—Survival curves for subgroups of patients who had undergone radical (a, b) or palliative (c, d) resection of their tumours. Subdivisions are based on ranges of preoperative serum CEA concentration (a, c) or TNM classification (b, d). The dotted curves represent the survival curves for all patients with radically resected (a, b) or palliatively resected tumours (c, d). The total number of patients in each subgroup and the statistical significance is listed in Table III.

TABLE III.—*Statistical significance of differences between the survival curves of related subgroups of patients as shown in Fig.* 3

| Graph | Subgroups of patients | | Age ratio (< 70/≥ 70) | $n_o$ | $n_p$ | $P$ |
|---|---|---|---|---|---|---|
| | Patients with radical resection | | | | | |
| (a) | Preoperative CEA | | | | | |
| | (μg/l) | 0–2 | 1·00 | 122 | 0 | 0·08 |
| | | 2–10 | 1·07 | 194 | 2 | < 0·001 |
| | | > 10 | 1·25 | 36 | 2 | |
| (b) | Tumour extension (TNM) | | | | | |
| | T1–2NoMo | | 1·13 | 66 | 0 | 0·4 |
| | T3 NoMo | | 1·03 | 130 | 2 | 0·02 |
| | T4 NoMo | | 0·91 | 82 | 1 | 0·08 |
| | T1–4N1–3Mo | | 1·28 | 64 | 0 | |
| | Patients with palliative resection | | | | | |
| (c) | Preoperative CEA | | | | | |
| | (μg/l) | 0–10 | 1·06 | 103 | 11 | < 0·001 |
| | | > 10 | 0·91 | 44 | 5 | |
| | | 0–5* | 1·21 | 73 | 8 | 0·018 |
| | | > 5* | 0·85 | 74 | 8 | |
| (d) | Tumour extension (TNM) | | | | | |
| | T1–4No–3Mo | | 1·22 | 100 | 12 | 0·02 |
| | T1–4No–3M1 | | 0·69 | 44 | 4 | |

* Not shown in Fig. 3.

the categories of resectability (*viz.* R = radical resection, P = palliative resection and NR = nonresectable) yielded survival curves (Fig. 1c) showing highly significant differences (Table I). Patients with nonresectable tumours had such a poor prognosis that further computations for this group examining other prognostic parameters were omitted. This group also exhibited a shift in the age distribution, with more patients ≥ 70 years (Table I).

Computations of the survival curves based on the criteria of tumour extension (TNM classification) could be performed only with 524 patients, since staging of 39 patients was incomplete. The survival curves of patients subdivided into 5 groups with different degrees of tumour extension are given in Fig. 1d. No significant differences were found between patients with T1–2NoMo tumours and patients with T3NoMo tumours. However, the survival curves were significantly different between patients with T3NoMo tumours and those with T4NoMo tumours. All cases with lymph-node metastasis showed a significantly lower survival which was also significantly different from those with distant metastasis. Patients with distant metastasis also showed more patients ≥ 70 years (Table I).

*Prognostic criteria based on combinations of two parameters*

To test whether the preoperative serum CEA level is a prognostic factor independent of degree of tumour extension, computations of the survival curves were performed for groups of patients with different tumour extension according to preoperative serum CEA levels. The survival curves of these subgroups are given in Fig. 2. The results showed significant differences between patients with CEA ranges of 0–5 μg/l and > 5 μg/l in the 4 classes of tumour extension, T1–2NoMo, T3NoMo, T4NoMo and T1–4N1–3Mo (Table II). We also gave the survival curves of patients with preoperative CEA levels > 10 μg/l, to illustrate the higher risk of these patients. In patients with distant metastasis (T1–4No–3M1) survival was not significantly dependent on preoperative CEA ranges between 0–5 and > 5 μg/l ($P = 0{\cdot}2$; Table II) possibly due to the greater malignancy of metastasizing tumours.

Survival curves based on the combination of the two prognostic parameters

FIG. 4.—Survival curves for subgroups of patients with radically resected T1–3NoMo tumours (a), T4NoMo tumours (b), and T1–4N1–3Mo tumours (c), and with palliatively resected T1–4No–3Mo tumours (d) according to serum CEA level (μg/l). The dotted curves are the survival curves for all patients registered in each of the 4 TNM subgroups. The number of patients in each subgroup and the statistical significance is listed in Table IV.

tumour extension and preoperative serum CEA level still included all patients with nonresectable tumours, who generally have a poor prognosis. Therefore, computations were then performed separately for patients with radically resected tumours and those undergoing palliative resection. Subgroups were selected based on different ranges of preoperative CEA. Furthermore, we examined the prognostic value of tumour extension combined with resectability. The resulting survival curves are shown in Fig. 3. In both the group of patients with radical resection and those with palliative resection, subgroups with distinct ranges of preoperative CEA could be established showing a significantly different survival (Table III) based on preoperative CEA ranges of 0–10 and > 10 μg/l, 0–5 and > 5, or 0–2 and 2–10 and > 10 μg/l. Similarly, significantly different survival curves were found in subgroups of patients with radical or palliative resection when different stages of tumour extension were considered (Table III). Not listed in the table is a single patient with distant metastasis (M1) who had undergone radical resection. These data indicate that all three parameters, resectability, tumour extension and preoperative serum CEA levels, are of prognostic value for patients, whether with radically or palliatively resected colorectal cancer. Survival of patients with nonresectable tumours revealed no dependence on preoperative serum CEA levels.

*Prognostic value of combinations of three parameters*

Final confirmation that the preoperative serum CEA level can be used as independent prognostic parameter was obtained from computations of survival of subgroups of patients with distinct resectability and distinct tumour extension. Patients who had undergone radical resection were subdivided into subgroups with the following tumour extensions: T1–3NoMo, T4NoMo and any tumours with lymph-node metastasis (T1–4N1–3Mo). T1–2NoMo and T3NoMo tumour stages were combined, since patients with these stages showed no significantly different survival (see Table III). A single patient with distant metastasis (M1) but radical resection was excluded. Patients who had undergone palliative surgery were represented by only one subgroup (T1–4No–3Mo) since survival of patients with distant metastasis (M1) was not significantly dependent on preoperative CEA levels (see Table II). The survival curves are shown in Fig. 4. The differences between survival curves based on preoperative CEA ranges of 0–5 and > 5 μg/l were significant for patients with radical resection and T1–3NoMo tumours (Table IV) but not for patients with radical resection and lymph-node metastasis ($P=$

TABLE IV.—*Statistical significance of differences between the survival curves of related subgroups of patients as shown in Fig.* 4

| Graph | | Preoperative CEA (μg/l) in subgroups of patients | Age ratio (< 70/≥ 70) | $n_o$ | $n_p$ | $P$ |
|---|---|---|---|---|---|---|
| | Patients with radical resection | | | | | |
| (a) | T1–3NoMo tumours | 0–5 | 1·14 | 154 | 1 | < 0·001 |
| | | > 5 | 0·82 | 42 | 1 | |
| (b) | T4NoMo tumours | 0–5 | 0·85 | 50 | 1 | 0·02 |
| | | > 5 | 1·70 | 27 | 0 | |
| (c) | T1–4N1–3Mo tumours | 0–5 | 1·04 | 45 | 0 | 0·1 |
| | | > 5 | 2·16 | 19 | 0 | |
| (d) | Patients with palliative resection and T1–4No–3Mo tumours | 0–10 | 1·23 | 75 | 7 | < 0·001 |
| | | > 10 | 1·36 | 25 | 5 | |

0·1) possibly due to the few patients in the subgroups with the CEA range $>5$ $\mu$g/l. Survival curves of patients who underwent only palliative resection of tumours with or without lymph-node metastasis (T1–4No–3Mo) revealed significant differences between preoperative CEA ranges of 0–10 and $>10$ $\mu$g/l (Table IV) but not between 0–5 and $>5$ $\mu$g/l ($P=0{\cdot}15$).

### DISCUSSION

The generally accepted prognostic criteria for tumour surgery are resectability, site of the tumour, tumour extension, age and general condition of the patient. A preliminary statistical analysis to examine the validity of these prognostic parameters, including the preoperative serum CEA level as a molecular marker, was performed with data collected during a long-term post-operative follow-up of 563 patients with colorectal cancer since 1974. All these potentially prognostic criteria were available within a short period of hospitalization for surgery.

The survival curves for various subgroups of patients opened up the possibility of comparing different prognostic parameters, and improving the prognostic information by combinations of 2 or more parameters. Resectability (using the criteria "radical resection", "palliative resection" and "nonresectable") was confirmed as a highly significant prognostic parameter. Considering the site of the tumours in colorectal cancer, it is generally assumed that prognosis improves with the site of the tumour, in the order rectum < sigmoid colon < ascending, transverse, descending colon and caecum. In our 563 patients the tumour sites did not represent a significant prognostic parameter. However, tumour extension had reliable prognostic value. Though the stages of localized tumour extension (T1–3NoMo) had little prognostic value when compared with each other, they showed a significantly improved survival over T4NoMo tumours. Prognosis became significantly worse when patients had already developed lymph-node metastasis or even distant metastasis. The age of patients at surgery implied prognostic significance for patients $<70$ and $\geqslant 70$ years. This finding has to take into account that prognostic criteria, such as nonresectability and distant metastasis as well as a generally poorer physical condition, are associated with age over 70 (see Table I) and could be responsible for the poorer prognosis.

The more important question to be answered in this report was whether a molecular marker (*i.e.* the preoperative serum CEA level) has prognostic value as a single parameter or in combination with other prognostic parameters. Differences in preoperative CEA ranges between 0–2 and 2–4 $\mu$g/l had no prognostic significance. However, differences between survival curves were significant for patients with CEA ranges of 2–4, 4–10 and $>10$ $\mu$g/l independent of other prognostic parameters such as resectability and tumour extension. Survival curves of subgroups of patients based on combinations of the preoperative CEA levels with a second and third prognostic parameter confirmed that distinct preoperative CEA levels can be independent prognostic markers. Exceptions were patients with very poor prognosis, mostly due to far-advanced tumour progression, *i.e.* patients with distant metastasis or nonresectable tumours, who had a very short survival time. In this group of patients the biological situation might be predominantly influenced by additional physiological disorders such as cachexia, which apparently do not affect the production and secretion of CEA by tumour cells.

Why the level of CEA secretion into the serum by tumour cells before surgery reflects the further development of the malignant disease, even after radical resection of the tumour, is little understood. A possible explanation might be the influence of circulating tumour antigens on immune surveillance as potential inducers of suppressor cells. Suppressor T cells responsive to tumour antigens prevent both the generation (Greene

*et al.*, 1977*a*, *b*) and expression (Asherson & Zembala, 1976) of T effector cells. These suppressor T cells persist after surgical removal of the tumour (Fujimoto *et al.*, 1976) whereas the direct blocking effect of tumour antigens and its immune complexes against T effector cells observed with tumour-bearer sera disappears within a few days after tumour removal (Hellström *et al.*, 1970). A second explanation may be a direct correlation of the preoperative serum CEA level with clinically undetectable micrometastasis responsible for the further development of the malignant disease.

The gain in prognostic information represented by distinct ranges of the preoperative serum CEA should facilitate the management of patients for adjuvant postoperative treatment such as chemotherapy or immunotherapy.

It can be expected that early adoption of postoperative treatment might improve the prognosis of patients. A generalization from our results has to be based on our methods. If other CEA test systems were used, different critical ranges of the preoperative CEA levels would be expected.

### REFERENCES

American Joint Committee for Cancer Staging and End Results Reporting (1977) *Manual for Staging of Cancer*. Chicago.

Asherson, G. L. & Zembala, M. (1976) Suppressor T-cells in cell-mediated immunity. *Br. Med. Bull.*, **32**, 158.

Evans, J. T., Mittelman, A., Chu, M. & Holyoke, E. D. (1978) Pre- and postoperative uses of CEA. *Cancer*, **42**, 1419.

Fujimoto, S., Greene, M. I. & Sehon, A. H. (1976) Regulation of the immune response to tumor antigens. I. Immune suppressor cell in tumour-bearing hosts. *J. Immunol.*, **116**, 791.

Goslin, R., Steele, G., MacIntyre, J. & 5 others (1980) The use of preoperative plasma CEA levels for the stratification of patients after curative resection of colorectal cancers. *Ann. Surg.*, **192**, 747.

Greene, M. I., Dorf, M. E., Pierres, A. & Benacerraf, F. B. (1977*a*) Reduction of syngeneic tumor growth by an anti I-J alloantiserum. *Proc. Natl Acad. Sci. U.S.A.*, **74**, 5118.

Greene, M. I., Fujimoto, S. & Sehon, A. H. (1977*b*) Regulation of the immune response to tumor antigens. III. Characterization of thymus suppressor factors produced by tumor-bearing hosts. *J. Immunol.*, **119**, 757.

Hansen, H. J., Lance, K. P. & Krupey, J. (1971) Demonstration of an ion-sensitive antigenic site on carcinoembryonic antigen using zirconyl phosphate gel. *Clin. Res.*, **19**, 143.

Hellström, I., Hellström, K. E. & Sjögren, H. O. (1970) Serum mediated inhibition of cellular immunity to methylcholanthrene-induced murine sarcomas. *Cell. Immunol.*, **1**, 18.

Holyoke, E. D., Chu, T. M. & Murphy, G. P. (1975) CEA as a monitor of gastrointestinal malignancy. *Cancer*, **35**, 830.

Kohler, J. P., Simonowitz, D. & Paloyan, D. (1980) Preoperative CEA level: A prognostic test in patients with colorectal carcinoma. *Am. Surg.*, **46**, 449.

Peto, R. & Peto, J. (1972) Asymptotically efficient rank invariant test procedures. *J. R. Statist. Soc. A*, **135**, 185.

Peto, R., Pike, M. C., Armitage, P. & 7 others (1976 and 1977) Design and analysis of randomized clinical trials requiring prolonged observation of each patient. I. Introduction and design. *Br. J. Cancer*, **34**, 585. II. Analysis and examples. *Br. J. Cancer*, **35**, 1.

Staab, H. J., Anderer, F. A., Stumpf, E. & Fischer, R. (1978) Slope analysis of the postoperative CEA time course and its possible application as an aid in diagnosis of disease progression in gastrointestinal carcinoma. *Am. J. Surg.*, **136**, 322.

Steele, G., Zancheck, N., Wilson, R. & 4 others (1980) Results in CEA-initiated second-look surgery for recurrent colorectal cancer. *Am. J. Surg.*, **139**, 544.

TNM Classification of Malignant Tumors (1978) Ed. Harmer. 3rd edn. New York: Springer-Verlag.

Wanebo, H. J., Rao, B., Pinsky, C. M. & 4 others (1978) Preoperative carcinoembryonic antigen level as a prognostic indicator in colorectal cancer. *N. Engl. J. Med.*, **299**, 448.

Wood, C. B., Ratcliffe, J. G., Burt, R. W., Malcolm, A. J. H. & Blumgart, L. H. (1980) The clinical significance of the pattern of elevated serum carcinoembryonic antigen (CEA) levels in recurrent colorectal cancer. *Br. J. Surg.*, **67**, 46.